# Activity Rhythms of Coexisting Red Serow and Chinese Serow at Mt. Gaoligong as Identified by Camera Traps

**DOI:** 10.3390/ani9121071

**Published:** 2019-12-02

**Authors:** Yixin Chen, Zhishu Xiao, Long Zhang, Xinwen Wang, Ming Li, Zuofu Xiang

**Affiliations:** 1 College of Life Science and Technology, Central South University of Forestry & Technology, Changsha 410004, China; phoenixwindrunner@aliyun.com (Y.C.); longzhang_csuft@163.com (L.Z.); 2 Institute of Evolutionary Ecology and Conservation Biology, Central South University of Forestry & Technology, Changsha 410004, China; 3 State Key Lab of Integrated Management of Pest Insects and Rodents, Institute of Zoology, Chinese Academy of Sciences, Beijing 100101, China; xiaozs@ioz.ac.cn; 4 Lushui Bureau of Mt. Gaoligong National Nature Reserve, Liuku 673229, China; wxw13988678557@126.com; 5 Key Lab of Animal Ecology and Conservation Biology, Institute of Zoology, Chinese Academy of Sciences, Beijing 100101, China; lim@ioz.ac.cn

**Keywords:** camera traps, activity rhythm, competition, montane forest, species interactions, niche partitioning, Bovidae, Gaoligong

## Abstract

**Simple Summary:**

How congeneric species with similar realized niches manage to coexist is a central question in the study of biodiversity. Here, we examined the daily activity rhythm of two coexisting serow species in a mid-mountain humid evergreen broadleaf forest. We used camera traps in a five-year survey at Mt. Gaoligong, western Yunnan, China. We compared the daily activity rhythm of the rare red serow (*Capricornis rubidus*), a medium-sized solitary ungulate, with the coexisting Chinese serow (*C. milneedwardsii milneedwardsii*). Although their overall daily activity rhythms were similar, the rare red serow tended to range, feed, and stay vigilant from afternoon through midnight throughout the year. By contrast, Chinese serows preferred to be active from sunrise to noon in the wet season, but shifted their activities and behaviors to afternoon and midnight in the dry season. Interestingly, we found red serows sometimes ranging together with Chinese serows. When they encountered each other, red serows altered their activity patterns more notably, while Chinese serows significantly increased their activity level. These findings are understandable given their similar resource requirements. Although exploitative competitors, red and Chinese serow coexist by avoiding interference competition by altering their respective activity patterns in time.

**Abstract:**

Surveying the activity rhythms of sympatric herbivorous mammals is essential for understanding their niche ecology, especially for how they partition resources and their mechanisms of coexistence. Over a five-year period, we conducted infrared camera-trapping to monitor the activity rhythms of coexisting red serow (*Capricornis rubidus*) and Chinese serow (*C. milneedwardsii milneedwardsii*) in the remote mountainous region of Pianma, Mt. Gaoligong, Yunnan, China. Cameras captured images of red serow and Chinese serow on 157 and 179 occasions, respectively. We used circular kernel density models to analyze daily activity rhythms and how temporal variations in activity ensure their co-existence. Although their overall activity levels and patterns were similar, temporal activity and behavior partitioning among the two species occurred during the wet season. Compared with Chinese serows, red serows exhibited less variable daily activity levels, patterns, as well as feeding and vigilance behaviors between seasons. When the two species occasionally ranged together, red serows tended to alter their activity pattern while Chinese serows significantly increased their activity level. Red serow and Chinese serow are exploitative competitors but coexist by altering their daily activity rhythms when in contact and changing activity patterns during the wet season, enabling their coexistence.

## 1. Introduction

Viewed from an evolutionary perspective, animal behavior should strategically balance resource acquisition [1]. An animal’s activity rhythm can be defined as “how an individual partitions its behavior over time” while balancing the effects of both abiotic and biotic factors [1,2]. Abiotic factors, such as temperature, rainfall, and light levels, may impact food availability, thermal regulation, and endogenous rhythm, resulting in changes to activity rhythms and time budgets [3,4,5,6,7,8]. Biotic factors, especially interactions among species, also play an important role [9,10,11,12]. According to the notions of limiting similarity and competitive exclusion, no two species can coexist in sympatry unless limited resources are partitioned adequately [13,14,15], namely, niche partitioning [1]. To reduce or avoid interference competition and predation, co-existing congeneric species with similar morphological and dietary traits may exhibit differences in their temporal use of resources [2,3,4,6,7,9,10,11,12,13,14,15,16]. 

Individuals of congeneric species may vary activity and behavior patterns over different timescales on a daily, weekly or seasonal basis to track the availability of resources while avoiding interference competition [1,13,14,15,17]. The latter has been observed among various animal guilds [4,12,18,19] including ungulates [6,9,10,11,16,20,21,22,23,24,25]. Moreover, when two species coexist or encounter each other, species with larger body size or social group are usually dominant over the smaller species, leading the latter to alter their activity and behavior patterns [6,11,24,25]. Thus, examining the activity rhythms among coexisting species is essential for understanding their behavioral ecology and the mechanisms by which species within animal guilds manage to coexist in forest ecosystems [4,6,7,9,10,11,12,18,19,20,21,22,23,24].

Serows *Capricornis* spp. (Caprinae, Tribe Caprini, Bovidae) are medium-sized ungulates inhabiting the rugged montane forests (ranging from 0 to 4500 m above sea level) of eastern and southeastern Asia [26,27,28,29]. Given the disputes in taxonomy, there are anywhere from four to seven sibling species existing in these forests, while distributions of some mainland species may overlap [26,27,28,29,30]. The near-threatened red serow (*Capricornis rubidus*) (body length: 140–155 cm; shoulder height: 85–95 cm; weight: 110–160 kg) is the most enigmatic serow species [26,27,28,29,30,31,32]. Its rarity has led to very little information about the basic biology and ecology of the species being collected, and almost nothing is known about its activity rhythms and behavioral patterns [28,29,31]. However, it is assumed that the red serow may exhibit similar ecological traits (e.g., social organization, diet, habitat utilization, activity and behaviors) to congeneric species such as the Chinese serow (*C. milneedwardsii*) [6,21,28,29,33,34,35,36], Himalayan serow (*C. thar*) [20,33,37,38], Japanese serow (*C. crispus*) [28,29,39,40,41], and Taiwan serow (*C. swinhoei*) [28,29,33,42]. Although red serow mainly occur in northern Myanmar [26,27,28,29], it is found also in eastern Assam, India [43], as well as western Yunnan, China in recent years [44,45]. Red serow co-exists with slightly-larger Chinese serow (*C. milneedwardsii milneedwardsii*) (body length: 140–180 cm; shoulder height: 100–112 cm; weight: 85–140 kg) in some forests along the China–Myanmar border [26,27,28,29,33,46], especially in the region of Mt. Gaoligong of western Yunnan [44,45,46,47]. The mechanisms by which these two congeneric and forest-dwelling ungulates co-exist in these montane forests remains unclear [26,27,48].

By conducting long-term infrared camera-trap surveys, we quantified the daily and seasonal activity rhythms and behaviors of sympatric red serow and Chinese serow as well as inferred temporal ways in which these two closely related species co-exist at Pianma, Mt. Gaoligong, western Yunnan, China. We hypothesized that (1) these two co-existing, congeneric species may exhibit differentiations in activity rhythms to reduce potential competition [1,2,13,14,15], (2) that the activity rhythms and behavior patterns of these two species may vary between different season [3,4,5,6,7,8], and (3) that the slightly-larger Chinese serow may lead red serow to exhibit obvious fluctuations in in activity rhythms when the two species encounter each other [7,11,24,25].

## 2. Methods

### 2.1. Study Site

**Figure 1:**
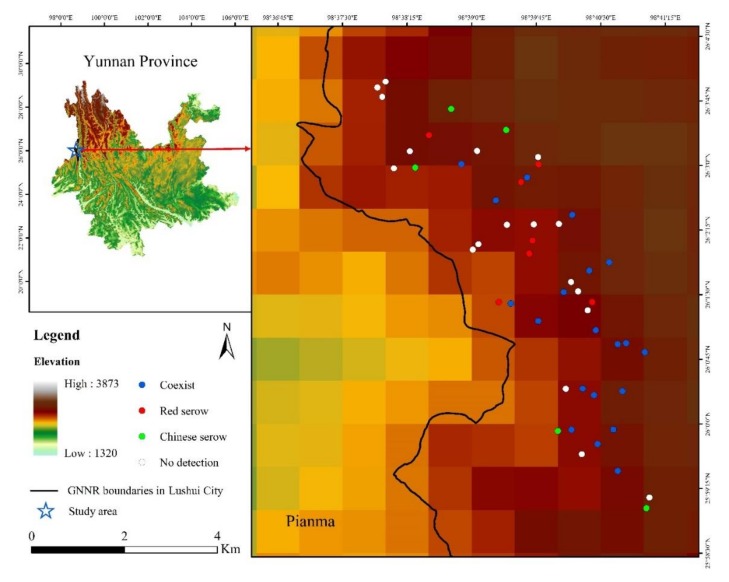
Study area and camera-trapping sites indicating where red and Chinese serows coexist. Coexisting (blue dot): both red serow and Chinese serow were detected; red serow site (red dot): only red serow detected; Chinese serow (green dot): only Chinese serow detected; no detection (white dot): sites where neither red serow nor Chinese serow were detected.

We conducted surveys at Pianma (26°2.337′ N, 98°39.127′ E), which is located on the western slope of the southern section of Mt. Gaoligong National Nature Reserve (GNNR, Figure 1), in Lushui City, Nujiang Lisu Autonomous Prefecture, Yunnan Province, China [47,49,50]. The survey area is considered a world-class biodiversity hotspot as well as in China [51,52,53], with very steep mountainous terrain ranging from 1900–3800 m a.s.l. [54]. Primary vegetation zonation at Pianma falls into three types: (1) mid-mountain humid evergreen broadleaf forest (2000–2800 m), (2) temperate conifer forest (2700–3100 m), and (3) cold bamboo–conifer mixed forest (3100–3800 m) associated with alpine meadow and shrubbery [47,49,55]. Mean annual temperature and mean annual precipitation vary with altitude, ranging from 13.59 to 2.97 °C and 1200–3900 mm [54]. The wet season (May to mid-October) provides 75–80% of annual precipitation [5,50,54], with mean monthly temperatures ranging from 16.87 (1900 m) to 6.25 °C (3800 m) [50,54]. In the dry season (late-October to late-April), mean monthly temperatures range from 10.32 (1900 m) to −0.30 °C (3800 m) [50,54]. Potential predators for serows at Pianma, such as tiger (*Panthera tigris*), leopard (*P. pardus*), clouded leopard (*Neofelis nebulosa*), Asiatic golden cat (*Catopuma temminckii*), grey wolf (*Canis lupus*) and dhole (*Cuon alpinus*), have not been reported for more than two decades [46,47]; thus, they are very likely to have vanished in the survey area. Traditionally, local residents occasionally go into the reserve for herb collecting, pasturing, selective logging or poaching [47,54,56], but human activities have decreased in recent years owing to strict management and law enforcement.

### 2.2. Infrared Camera-Trapping

As a part of the long-term monitoring of terrestrial mammals and birds in the region [47,49], we conducted five periods of infrared camera-trapping from 18 November 2013 to 27 January 2019 at Pianma. Based on local knowledge and accessibility of the terrain, we successively deployed 20 Ltl-Acorn 5210A, 10 Ltl-Acorn 6210MC and 30 Ltl-Acorn 6511MC cameras at 50 different sites within the reserve (Figure 1, Table 1) [47]. These cameras featured with nearly identical triggering and shooting functionalities, while the major differences were the latter two equipped better weather sealings and video format (1080 p). All cameras were able to take normal polychrome images (daytime) or infrared monochrome (nighttime or low environmental illumination) images when motion was detected. Generally, we attached cameras to trees, at a height of 30–50 cm, facing animal tracks, water sources, mineral licking sites, and resting sites at altitudes ranging from 2570–3447 m (Table 1). Neighboring cameras were deployed at different altitude or ecotype (different vegetation types) and spaced 200–1000 m apart. We did not apply scent lures or baits at any camera-trapping sites. We tried to avoid deploying cameras near any locations with potential human activities (e.g., field tracks occasionally used by local residents for herb collecting). We adjusted detailed camera-trapping sites between each period based on the monitoring results of the previous period (Table 1) [47]. 

Camera settings were as follows: (1) photo and video (Ltl-Acorn 6511MC and Ltl-Acorn 6210MC) or photo (Ltl-Acorn 5210A); (2) highest image quality (12 megapixels for photo, 1080 px for video); (3) medium trigger sensitivity; (4) 1 s trigger interval; (5) three shots and one video (15 s) per trigger (Ltl-Acorn 6511MC and Ltl-Acorn 6210MC) or three shots only (Ltl-Acorn 5210A); (6) side prep sensors “on”; and (7) time and date stamp (China Standard Time CST, UTC + 8, central meridian 120°E) [47,49]. We used 16 GB SanDisk SDHC cards to record image data and 12 AA batteries to power the cameras. We checked cameras every 3–4 months over the five-year period of survey for retrieving SD cards and replacing batteries or malfunctioned cameras [47,49].

### 2.3. Data Analysis

#### 2.3.1. Identification of Serows

**Figure 2:**
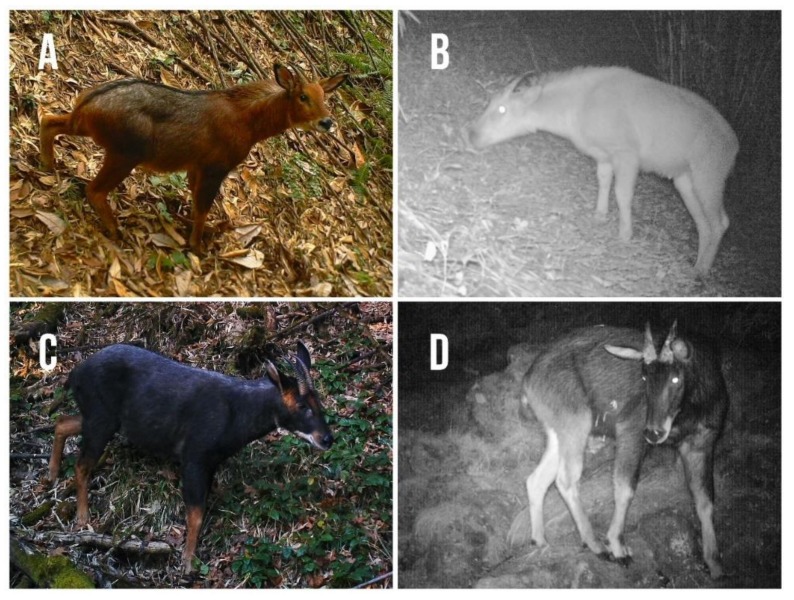
Polychrome and monochrome images of both red serow (*Capricornis rubidus*, **A**,**B**) and Chinese serow (*C. milneedwardsii milneedwardsii*, **C**,**D**) captured by infrared camera traps at Pianma, Yunnan, China from November 2013 to January 2019.

We carefully examined all images (photos and videos) captured by camera traps to identify serows. Where necessary, we used Adobe Photoshop CC 2017 and Premiere CC 2017 to enhance details on images to assist with species identification [47,49]. Both red serow and Chinese serow were easily distinguished from their pelage and throat patch color (Figure 2, Table 2) [26,27,28,29,33]. When vegetation, body orientation or blurred images precluded clear identification, we categorized uncertain serow “*Capricornis* spp.”. In addition, we uploaded image data into an online database for assisting in identification and categorization [57,58].

#### 2.3.2. Independent Detections and Behaviors 

We extracted independent detections of red serow, Chinese serow, and uncertain serow *Capricornis* spp. from image data acquired during the survey. We defined an independent detection as consecutive images of single serow species taken more than 30 min apart [6,47,49,59]. 

Considering that serows use a variety of habitats [6,26,27,28,29,33,35,37,38,42,43] and abiotic factors, such as temperature and precipitation, are highly associated with seasonal variation at Mt. Gaoligong [5,50,54], we only examined seasonal variation in activity rhythms. Thus, for season-specific comparisons, we pooled independent detections into the wet season (1 May–15 October) and the dry season (16 October–30 April) [5,50,54].

We also extracted co-occurring detections where both red serow and Chinese serow simultaneously appeared in the same independent detections, or in temporally-adjacent independent detections within 30 min at the same camera-trapping site. By contrast, we categorized and defined single-species detections as independent detections of one species alone.

Serows are generally solitary and territorial ungulates usually exhibiting primitive behaviors [26,27,28,29,33,40,41,42,60,61]. In order to explore the mechanisms of behavior partitioning of two congeneric serows in different seasons, we adopted all-occurrence sampling to record the occurrences of two fundamental behaviors [62,63], feeding and vigilance, from independent detections of each species, respectively. We defined feeding behaviors as serow browsing plants, drinking water or licking minerals [36,60]. Meanwhile, we defined vigilance behaviors as serow exhibiting freezing posture (i.e., standing still or lying low) or scanning surroundings vigilantly [40,41,61,64,65]. If serows exhibited vigilance or abnormal behaviors by firstly noticing the presence of camera traps, we excluded these records from our data set [66,67]. We did not record social behaviors due to very limited sample size (<10 independent detections).

Given our survey area was approximately 8°E of UTC + 6 (central meridian 90°E), we corrected time stamps of all independent detections to local time (UTC + 6.58) [68].

#### 2.3.3. Circular Kernel Density Models

Theoretically, the time stamp of independent detections can be treated as random sampling from continually-distributed time, following a circular von Mises distribution [69,70,71]. Thus, we fitted non-parametric circular kernel density models using the packages “*activity*” (Version 1.1) and “*overlap*” (Version 0.3.2) [72,73], in the statistical software R (version 3.6.1) [74]. In non-parametric circular kernel density models, activity rhythm can be separated into activity level and activity pattern [71,72]. Activity level is the ratio of the areas under and above the curve of the circular probability density function f(x), representing percentage of time active, while activity pattern is the shape and trend of the curve [71,72]. We first quantified overall activity levels and overlapping patterns by pooling all independent detections for both species. Then we analyzed season-specific activity levels, patterns and coefficients of overlapping in intra- and inter-species comparisons. Subsequently, we analyzed seasonal variations on feeding and vigilance behaviors. Finally, we compared the co-occurring detection set with single-species detection sets for differences in daily activity rhythms.

Initially, we converted local time stamps of independent detections into radian units [71,72,73], and further converted them into solar-time to account for astronomical events (e.g., variation of sunrise and sunset throughout year) using the package “*overlap*” [73,75]. Based on solar-time data, we used the package “*activity*” to fit circular kernel density models for each species to estimate their activity level with 10,000-times smoothed bootstrapping [72]. We subsequently carried out the randomization test and Wald test to detect differences in activity pattern and activity level between the two species [71,72]. 

Next, we estimated the coefficient of overlapping Δ (ranging from 0 to 1, the value close to 1 indicating complete overlap in activity) between two corresponding sets using the package “*overlap*” [69,73,76]. Given the adequate sample size, we adopted the Δ_4_ estimator (>50 independent detections for the smaller sample set) or Δ_1_ estimator (<50 independent detections for the smaller sample set) [69,73]. We also generated 10,000 times smoothed bootstrapping to estimate the confidence interval (CI) and mean value of the Δ_4_ estimator or Δ_1_ estimator. To avoid potential overflow in the range (from 0 to 1) of CI values, we carried out the corrections on a logistic scale and back transformation [73]. 

Based on sample size, we set the bandwidth adjustment factor as 1.5 or 1 during smoothed bootstrapping analysis to improve the performance of circular kernel density models by reducing biases [71]. We set significance level at 0.05 for the randomization test and Wald test. All scripts of R codes for analyses can be found in Supplementary Materials (File S1).

## 3. Results

### 3.1. Survey Results

We monitored 50 camera-trapping sites for a total 31,242 trapping days (Figure 1). While both serow species were mostly observed on their own (Table 3), at 54% (red serow) and 50% (Chinese serow) of the camera-trapping sites, they were also observed together at the same sites on thirteen occasions (3.9% of total independent records). Both species were detected at the same 20 camera-trapping sites (Table 3).

### 3.2. Overall Daily Activity Rhythm

**Figure 3:**
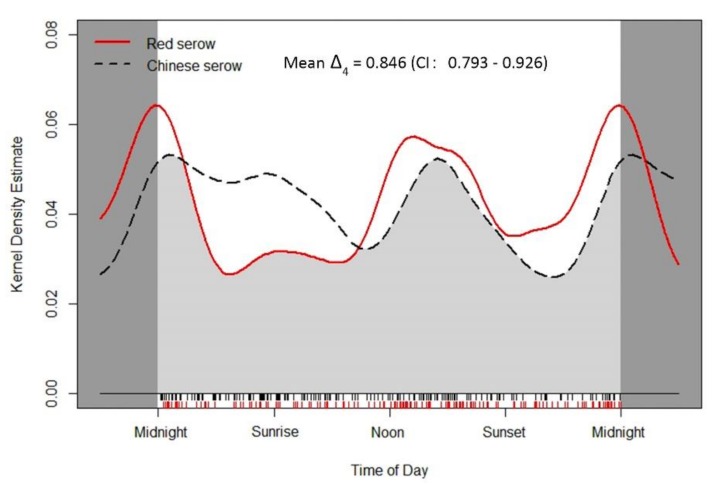
Circular kernel density models showing overall daily activity patterns of both red serow *Capricornis rubidus* (red, solid line) and Chinese serow *C. milneedwardsii milneedwardsii* (black, dashed line). The mean value of coefficient of overlapping Δ_4_ is represented by the light grey area under the curves. The small vertical bars under the grey area are independent detections.

Circular kernel density models indicated largely similar daily activity patterns between red serow and Chinese serow (Figure 3). Both species tended to be active in the afternoon and middle of the night, but Chinese serow appeared to be active from midnight through to mid-morning too. Both the randomization test and Wald test showed no significant differences of activity pattern (*p* = 0.119) and activity level (W = 1.476 ± 0.101; *p* = 0.224) between the two species (Table 4), and there was a significant level of overlap in their activity patterns (mean Δ_4_ = 0.846; CI: 0.793–0.926) (Figure 3). 

### 3.3. Seasonal Variation of Daily Activity Rhythms

**Figure 4:**
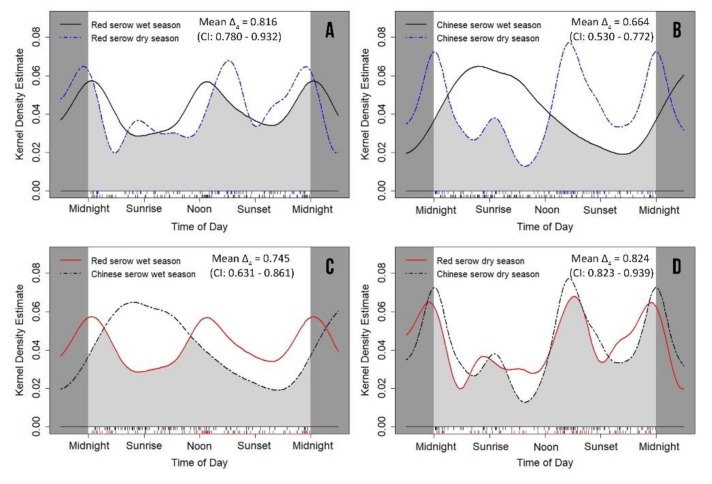
Circular kernel density models showing seasonal variation on daily activity patterns of both red serow *Capricornis rubidus* (**A**), Chinese serow *C. milneedwardsii milneedwardsii* (**B**), and their inter-species differences (**C**,**D**). The mean value of coefficient of overlapping Δ_4_ is represented by the light grey area under the curves. The small vertical bars under the grey area are independent detections.

Red serow’s daily activity levels (*W* = 0.222 ± 0.113; *p* = 0.637) and patterns (*p* = 0.382) between the wet and dry seasons were not significantly different (Figure 4A; Table 4 and Table 5), with high-level overlap between the seasonal patterns (mean Δ_4_ = 0.816; CI: 0.780–0.932) (Figure 4A). By contrast, Chinese serows were significantly more active around sunrise in the wet than the dry season (*p* < 0.0001) (Figure 4B; Table 4 and Table 5) and the overlap in activity pattern between the two seasons (mean Δ_4_ = 0.664; CI: 0.530–0.772) was much less than observed for red serow (Figure 4B).

Similarly, the daily activity patterns of red serow and Chinese serow in the wet season were significantly different (*p* = 0.002), though daily activity levels were not (*W* = 0.014 ± 0.110; *p* = 0.905) (Figure 4C; Table 4). However, daily activity patterns (*p* = 0.678) and levels in consecutive dry seasons (*W* = 0.618 ± 0.104; *p* = 0.432) were not different between serow species (Figure 4D; Table 4). Consequently, the overlap in activity pattern between the two species in the wet season was much less than in the dry season (Figure 4C,D).

### 3.4. Seasonal Variation of Feeding and Vigilance Behaviors

**Figure 5:**
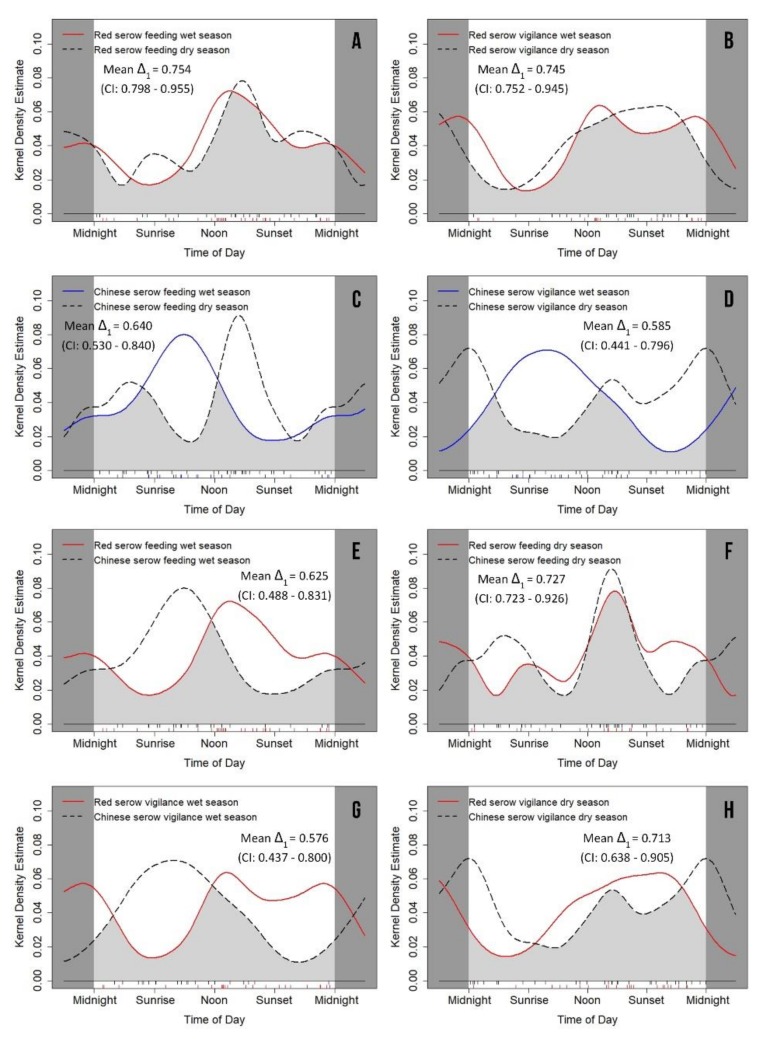
Circular kernel density models showing seasonal variation on feeding and vigilance behaviors of both red serow *Capricornis rubidus* (**A**,**B**), Chinese serow *C. milneedwardsii milneedwardsii* (**C**,**D**), and their inter-species differences (**E**–**H**). The mean value of coefficient of overlapping Δ_1_ is represented by the light grey area under the curves. The small vertical bars under the grey area are independent detections.

Generally, red serow tended to feed and be vigilant from noon to midnight (Figure 5A,B) and maintained stable feeding (*p* = 0.894) and vigilance (*p* = 0.751) patterns between the wet and the dry seasons (Table 5). By contrast, Chinese serow preferred to feed and be vigilant from sunrise to noon in the wet season, shifting their feeding and vigilance behaviors to afternoon and midnight during the dry season (Figure 5C,D). Thus, Chinese serow exhibited significant seasonal variations in feeding (*p* = 0.046) and vigilance behavior patterns (*p* = 0.014), and showing less overlap in activity patterns between seasons than red serow (Table 5; Figure 5A–D). 

The inter-species comparisons also indicated significant differences in feeding (*p* = 0.034) and vigilance behaviors (*p* = 0.017) patterns in the wet season (Figure 5E,G; Table 4). However, there were many more similarities between red serow and Chinese serow in feeding (*p* = 0.885) and vigilance (*p* = 0.380) behavior patterns in the dry season (Figure 5F,H; Table 4), resulting in more overlap in the dry season than in the wet season (Figure 5E–H). 

We did not detect significant differences on activity levels for feeding and vigilance behaviors in intra- and inter-species comparisons (Table 4 and Table 5).

### 3.5. Coexistence and Associated Daily Activity Rhythms

**Figure 6:**
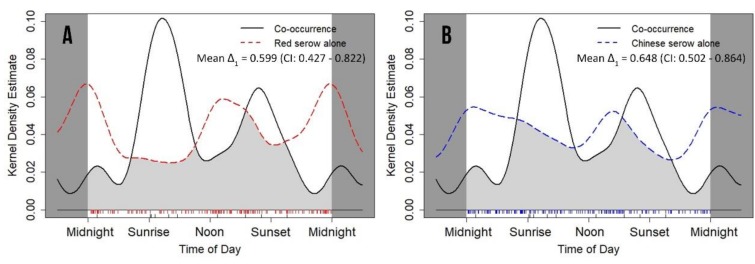
Daily activity patterns and overlapping comparisons between co-occurring detection (black, solid line) and single-species detections for red serow (**A**, red, dashed line) and Chinese serow (**B**, blue, dashed line). The mean value coefficient of overlapping Δ_1_ is represented by the light grey area under the curves. The small vertical bars under the grey area are independent detections.

There was a strong tendency towards statistical significance in the daily activity pattern in red serows when they were on their own and when with Chinese serows (*p* = 0.052) (Table 6) with lower overlap in activity pattern (mean Δ_1_ = 0.599; CI: 0.427–0.822) (Figure 6A). Similarly, Chinese serows did not change their activity pattern dramatically whether on their own or co-habiting with red serows (*p* = 0.149) (Table 6). However, they significantly changed their activity level when co-habiting with red serows (*W* = 5.689 ± 0.126; *p* = 0.017) (Table 6). Overlap in activity between the latter two states was greater (mean Δ_1_ = 0.648; CI: 0.502–0.864) (Figure 6B) than observed in red serow.

## 4. Discussion

Usually, temporal niche partitioning is very likely occurring between congeneric species occupying the same habitat [1,2,13,14,15,17]. Given the assumed biological and ecological similarities between red serow and Chinese serow [26,27,28,29,30,31,33,43,48], it was reasonable to expect they exhibiting similar resource demands in diets and habitat utilization in our survey area, which may intensify competition between these co-existing ungulates [1,13,14,15]. Our findings indicated significant differentiations in activity rhythms and behavior patterns between two congeneric serow species in the wet season (Figure 4C and Figure 5E,G; Table 4), suggesting temporal niche partitioning between two potential competitors [2,17]. Similar partitioning mechanisms also occur within other ungulate guilds [6,9,10,16,21,23,25], especially those coexisting congeneric species, such as native collared peccary (*Pecari tajacu*), white-lipped peccary (*Tayassu pecari*) and invasive feral pig (*Sus scrofa*) [7,22], red brocket deer (*Mazama americana*) and gray brocket deer (*M. gouazoubira*) [11], and Himalayan goral (*Nemorhaedus goral*) and Himalayan serow (*Capricornis thar*) [20]. 

However, potential competition between red serow and Chinese serow might not be sufficiently intensive to create strict inter-species avoidance [7,9,10,20,22,25], because they exhibited a high level overlaps in activity rhythms and behavior patterns in both the dry season and overall scale (Figure 3 and Figure 5F,H; Table 4). It could be attributed to solidarity and territoriality of serows [28,29,33,40,41,60], low population density or the abundance of potential food resources in Mt. Gaoligong [46,55], which provided sufficient resources to ensure their co-existence [1].

Seasonal variation in activity rhythm is an adaptive strategy to respond to seasonal changes in resources [3,4,5,6,7,8]. It can be found in various ungulate species, for example, blue sheep (*Pseudois nayaur*) [3], red deer [8], Alpine chamois (*Rupicapra rupicapra rupicapra*) and mouflon (*Ovis gmelini musimon* × *Ovis* sp.) [23]. However, in our study, red serows did not exhibit significant variations in their daily activity rhythms, feeding and vigilance behaviors between the wet and dry season Figure 4A and Figure 5A,B; Table 4 and Table 5). By contrast, such seasonal variations in Chinese serow were marked (Figure 4B and Figure 5C,D; Table 4 and Table 5). Chinese serows obviously preferred to be more active around sunrise in the wet season than in the dry season (Figure 4B and Figure 5C,D). These outcomes suggest that red serows might be “generalists” who are more capable to cope with seasonal changes in environment factors (e.g., rainfall, temperature or habitat type) than Chinese serows [77,78]. However it might also implicate latent food preference of Chinese serows [36,42,79]. In addition, the activity rhythm of Chinese serows in our survey area was similar to another population in Rini sacred mountain of Deqin county, Yunnan Province [21], where they were also active in afternoon. However in some areas of Shaanxi and Sichuan Provinces, Chinese serows were largely nocturnal [6,34]. These discrepancies in activity rhythm were likely owing to variations in habitat types, climate conditions, food resources, relations with other species or human disturbances between different areas [6,21,34].

Serows were known to co-exist with other ungulate species [6,20,21,34,42,47,79,80], but very little was known about how they reacted when they encountered other species [27,28,29]. In the present study, red serows and Chinese serows occasionally ranged together. When ranging together, both species altered their daily activity patterns and levels in different ways. Despite insufficient support in statistics due to limited sample size, red serows exhibited strong tendency to dramatically alter their daily activity patterns than Chinese serows did, while Chinese serows significantly increased their daily activity level when they ranging together. Given the similar body size, solidarity and other ecological traits [26,27,28,29,30,31,33,43,48], it suggested no clear dominance between these two species, unlike other ungulate guilds [7,9,10,11,16,24,25]. Ranging together could be a potentially useful anti-predator strategy [65], benefiting the survival of generally solitary serows. In addition, the two co-existing species clearly encountered each other and interacted often enough for possible interbreeding and hybridization to occur [27]. Generally, ranging together of red serows and Chinese serows was rare and had not been clearly reported previously [27]. Further research is required to clarify these complex behavioral inter-relationships. 

## 5. Conclusions

Despite similar overall activity levels and patterns, temporal niche partitioning between the two serow species is clear in the wet season. Red serows’ daily activity levels patterns, feeding and vigilance behaviors are less variable between seasons than in Chinese serows. Seasonal adjustments in daily activity and behaviors are greater by Chinese serow. Lastly, when the two species interact, they both alter their activity rhythms in different ways, especially Chinese serow. Our findings implicate temporal niche-partitioning mechanisms for these co-existing, elusive forest-dwelling ungulates, which were essential to enrich rudimentary knowledge of these poorly known and threatened species. Without camera traps, such information would have been logistically difficult to obtain, particularly without significant resources in such rugged montane forest.

## Figures and Tables

**Table 1:** Infrared camera-trapping efforts at Pianma from November 2013 to January 2019.

Trapping Periods	Camera-Trapping Sites	Trapping Days	Elevation (m)
November 2013 to November 2014	29	4298	2570–3240
November 2014 to October 2015	24	5039	2570–3425
October 2015 to October 2016	20	4053	2620–3425
November 2016 to November 2017	32	9827	2570–3447
November 2017 to January 2019	26	8025	2620–3447
Total camera-trapping sites: 50		Total trapping days: 31,242	

**Table 2:** Morphological traits used for identification of serows.

Trait	Red Serow (*Capricornis Rubidus*)	Chinese Serow (*C. Milneedwardsii Milneedwardsii*)
Overall coloration	Reddish-brown	Black, dark brown
Overall coloration in monochrome images	Light grey	Black, dark grey
Hair	Slick, very short	Coarse rather thin
Mane	Very short, dark red	Medium, mixed black to pale yellow
Jaw streak	White	White to golden-brown
Throat patch	Large and continuous, white	Usually discrete, white
Upper half of legs	Reddish-brown	Black
Lower half of legs	Buffy red	Reddish tan, creamy white

**Table 3:** Summary of monitoring results for serows at Pianma from November 2013 to January 2019. The number of photos and videos were the total number of each type of images triggered by the passing serows. Unless otherwise indicated, the values from 4 to 12 rows represent the number of independent camera-trap detections.

Category	Red Serow (*Capricornis Rubidus*)	Chinese Serow (*C. Milneedwardsii Milneedwardsii*)	Uncertain Serow (*Capricornis* Spp.)
Number of photos	956	1125	106
Number of videos	263	225	36
Camera-trapping sites	27	25	3
Independent detections	157	179	7
Detections in wet season	82	86	0
Detections in dry season	75	93	7
Feeding in wet season	31	23	0
Feeding in dry season	20	35	0
Vigilance in wet season	22	21	0
Vigilance in dry season	23	31	0
Co-occurring detections	13	13	0
Single-species detections	144	166	0

**Table 4:** Estimated overall and seasonal daily activity levels of red serow (*Capricornis rubidus*) and Chinese serow (*C. milneedwardsii milneedwardsii*), and results of inter-species comparisons. The estimates represent daily activity levels, i.e., the ratio of the areas under and above the curve of the circular probability density function f(x). The lower-case “w” and “d” in round brackets represent wet season and dry season. R. Test represents randomization test. The asterisks (*) represent significant differences.

Category	Red Serow (*Capricornis Rubidus*)		Chinese Serow (*C. Milneedwardsii Milneedwardsii*)		R. Test	Wald Test	
	Estimate	CI	Estimate	CI	*p*	*W*	*p*
Overall	0.573 ± 0.074	0.449–0.737	0.696 ± 0.069	0.526–0.795	0.119	1.476 ± 0.101	0.224
Wet season	0.592 ± 0.082	0.342–0.666	0.605 ± 0.073	0.380–0.665	0.002 *	0.014 ± 0.110	0.905
Dry season	0.538 ± 0.077	0.324–0.623	0.457 ± 0.070	0.287–0.560	0.678	0.618 ± 0.104	0.432
Feeding (w)	0.475 ± 0.094	0.223–0.588	0.449 ± 0.095	0.219–0.581	0.034 *	0.039 ± 0.133	0.844
Feeding (d)	0.461 ± 0.101	0.208–0.598	0.380 ± 0.089	0.217–0.561	0.885	0.359 ± 0.135	0.549
Vigilance (w)	0.517 ± 0.105	0.203–0.602	0.530 ± 0.090	0.209–0.557	0.017 *	0.009 ± 0.138	0.926
Vigilance (d)	0.563 ± 0.095	0.236–0.604	0.478 ± 0.108	0.234–0.645	0.380	0.350 ± 0.143	0.554

**Table 5:** Results of intra-species’ comparisons on seasonal variations of activity patterns and activity levels for red serow (*Capricornis rubidus*) and Chinese serow (*C. milneedwardsii milneedwardsii*). The R. Test represents randomization test. The lower-case “w” and “d” in round brackets represent wet season and dry season. The asterisks (*) represent significant differences.

Category	Red Serow (*Capricornis Rubidus*)			Chinese Serow (*C. Milneedwardsii Milneedwardsii*)		
	R. Test	Wald Test		R. Test	Wald Test	
	*p*	*W*	*p*	*p*	*W*	*p*
Wet season/Dry season	0.382	0.222 ± 0.113	0.637	<0.0001 *	2.148 ± 0.101	0.143
Feeding (w)/Feeding (d)	0.894	0.011 ± 0.138	0.917	0.046 *	0.283 ± 0.130	0.595
Vigilance (w)/Vigilance (d)	0.751	0.107 ± 0.141	0.744	0.014 *	0.135 ± 0.141	0.713

**Table 6:** Estimated daily activity levels of single-species detections for red serow (*Capricornis rubidus*), Chinese serow (*C. milneedwardsii milneedwardsii*), the co-occurring detection, and the results of randomization tests and Wald tests. The estimates represent daily activity levels, i.e., the ratio of the areas under and above the curve of the circular probability density function f(x). R. Test represents randomization test. The asterisk (*) represents significant difference.

Category	Single-Species		Co-Occurring		R. Test	Wald Test	
	Estimate	CI	Estimate	CI	*p*	*W*	*p*
Red serow (*Capricornis rubidus*)	0.547 ± 0.073	0.431–0.715	0.383 ± 0.103	0.147–0.548	0.052	1.590 ± 0.127	0.207
Chinese serow (*C. milneedwardsii milneedwardsii*)	0.683 ± 0.071	0.517–0.795			0.149	5.689 ± 0.126	0.017 *

## Supplementary Materials

The following are available online at https://www.mdpi.com/2076-2615/9/12/1071/s1, File S1: R codes and data sheets.
